# How Much of CAM Is Based on Research Evidence?

**DOI:** 10.1093/ecam/nep044

**Published:** 2011-06-18

**Authors:** Edzard Ernst

**Affiliations:** Complementary Medicine, Peninsula Medical School, Universities of Exeter and Plymouth, Exeter, UK

## Abstract

The aim of this article is to provide a preliminary estimate of how much CAM is evidence-based. For this purpose, I calculated the percentage of 685 treatment/condition pairings evaluated in the “Desktop Guide to Complementary and Alternative Medicine” which are supported by sound data. The resulting figure was 7.4%. For a range of reasons, it might be a gross over-estimate. Further investigations into this subject are required to arrive at more representative figures.

## 1. Introduction

A lively discussion exists about the question as to how much of conventional medicine might be based on sound evidence [1]. One figure that is often cited is 15% [2]. It presents, however, unreliable and out-dated information: the figure can be traced back to a small survey conducted in 1960/61 of prescribing practises of family doctors in a northern British town, which looked toward controlling prescribing costs [3]. Other experts have published more convincing data showing that an average of 76% of interventions are supported by some form of compelling evidence, with an average of 37% of interventions being supported by randomized clinical trials (RCTs) [3]. A recent systematic review [4] of the topic found that, in general internal medicine, over 50% [5] and in psychiatry over 65% [6] of interventions are based on positive data from RCTs.

The discussion about the evidence-base of CAM is far less lively. Here I present a first attempt to generate some data and hopefully a constructive discussion on this potentially important subject.

## 2. Methods

As a basis for my assessment, I used our own book *The Desktop Guide to Complementary and Alternative Medicine* [6]. In this book, we evaluate the research evidence from clinical trials and systematic reviews as it pertains to any type of CAM for a wide range of conditions (*n* = 46). For each condition, we compiled a “summary of clinical evidence” table in which the treatments are categorized according to the “weight” and “direction” of the evidence. The “weight” is conceptualized as a composite measure of the quantity, quality and level of the research evidence, which refers to the confidence that can be placed on that evidence [6]. The quantity refers to the total patient sample included in all clinical trials—there could, for instance, be five studies with an average of 20 patients resulting in a total sample of 100; this would be less than a single study with a sample of 300. The quality of the trial evidence refers to the likelihood of bias, usually estimated with a score such as the Jadad score [7]. The level of the evidence refers to the hierarchy of research evidence where systematic reviews are on top and opinion or anecdotal evidence at the bottom. The “direction” of the evidence signals whether the effect is clearly positive, tentatively positive, uncertain, tentatively negative or clearly negative [6]. The book has a full methods section to maximize transparency and reproducibility. It describes our assessments in more detail [6].

For the purpose of this analysis, I have simply counted the number of treatments which obtained the maximum “weight” and also were rated as “clearly positive” in our “summary of clinical evidence” tables. This provided the number of treatments that are supported by good evidence (if one therapy was effective for two indications it was counted twice). Subsequently, this figure was put in relation to the total number of treatment/condition pairings from all the “summary of clinical evidence” tables in our book [6].

## 3. Results

Fifty-one treatments were characterized as having maximum “weight” of evidence as well as being “clearly positive.” The total number of treatment/condition pairings was 685. Consequently, 7.4% of them were based on sound evidence.  Table 1 provides a list of these 51 treatment/condition pairings.

## 4. Discussion

The estimate that 7.4% of CAM is based on sound evidence may well be over-optimistic. We selected the conditions for inclusion in our book [6] on the basis of two main criteria: first, the condition had to be relevant, that is, commonly seen in primary care or frequently treated with CAM and/or there had to be sufficient trial data to write a chapter. Thus, this evidence summarized in the present article represents a positive selection. Had we chosen different conditions for our book, the percentage would most likely have been lower.

A glance at Table 1 furthermore informs us that several of the included modalities, for example, exercise, group behaviour therapy, stress management, fiber intake or biofeedback, could easily be classified as conventional interventions rather than CAM. Had we excluded them, the percentage of evidence-based CAM would have declined further.

Finally, several cases of “sound” evidence included in Table 1 might need revision in the light of evidence that has emerged since the publication of our book. Examples include saw palmetto (*Serenoa repens*) [8], glucosamine [9–12], *Ginkgo biloba* [13–16] and acupuncture which, according to recent findings, may not be more efficacious than sham acupuncture [17, 18].

Another concern is that the present analysis merely relates to the question of how many therapies might be supported by sound research evidence. It does not address the question of how solidly CAM practice is evidence-based. This would require an assessment of which treatments are used and how often. Such a research project would be complex but would certainly be a valuable contribution to the literature.

Although my estimate of how much of CAM is evidence-based draws on a critical evaluation of the available evidence, it still presents a rather optimistic view. Further investigations into this subject are required to arrive at more representative figures.

## Figures and Tables

**Table 1:** CAM treatments based on sound evidence.

Intervention	Conditions
Acupuncture	Nausea/vomiting induced by chemotherapy
Acupuncture	Osteoarthritis
African plum	Benign prostatic hyperplasia
Allium vegetables	Cancer prevention
Aromatherapy/massage	Cancer palliation
Biofeedback	Hypertension
Biofeedback	Migraine
Chondroitin	Osteoarthritis
Co-enzyme Q10	Hypertension
Diet	Rheumatoid arthritis
Ephedra sinica	Overweight
Exercise	Cancer prevention
Exercise	Cancer palliation
Exercise	Chronic fatigue syndrome
Exercise	Depression
Exercise	HIV/AIDS
Fiber	Irritable bowel syndrome
Ginkgo biloba	Alzheimer's disease
Ginkgo biloba	Peripheral vascular disease
Glucosamine	Osteoarthritis
Green tea	Cancer prevention
Group behaviour therapy	Smoking cessation
Guar gum	Diabetes
Guar gum	Hypercholesterolemia
Hawthorn	Chronic heart failure
Horse chestnut	Chronic venous insufficiency
Hypnotherapy	Labor pain
Kava	Anxiety
Massage	Anxiety
Melatonin	Insomnia
Music therapy	Anxiety
Oat	Hypercholesterolemia
Padma 28	Peripheral vascular disease
Peppermint/caraway	Non-ulcer dyspepsia
Phytodolor	Osteoarthritis
Phytodolor	Rheumatoid arthritis
Psyllium	Constipation
Psyllium	Diabetes
Red clover	Menopause
Relaxation	Anxiety
Relaxation	Insomnia
Relaxation	Nausea/vomiting induced by chemotherapy
*S*-adenosylmethionine	Osteoarthritis
Saw palmetto	Benign prostatic hyperplasia
Soy	Hypercholesterolemia
St John's wort	Depression
Stress management	HIV/AIDS
Tomato (lycopene)	Cancer prevention
Vitamin C	Upper respiratory tract infection (treatment)
Water immersion	Labor pain
Yohimbine	Erectile dysfunction
